# Coupling between cerebral blood flow and cerebral blood volume: Contributions of different vascular compartments

**DOI:** 10.1002/nbm.4061

**Published:** 2019-01-18

**Authors:** Roman Wesolowski, Nicholas P. Blockley, Ian D. Driver, Susan T. Francis, Penny A. Gowland

**Affiliations:** ^1^ Sir Peter Mansfield Imaging Centre University of Nottingham Nottingham UK; ^2^ Medical Physics and Imaging University Hospitals Birmingham NHS Foundation Trust Birmingham UK; ^3^ Wellcome Centre for Integrative Neuroimaging, FMRIB, Nuffield Department of Clinical Neurosciences University of Oxford Oxford UK; ^4^ Cardiff University Brain Research Imaging Centre, School of Psychology Cardiff University Cardiff UK

**Keywords:** arterial spin labelling, BOLD, CBF‐CBV coupling, cerebral blood volume, fMRI

## Abstract

A better understanding of the coupling between changes in cerebral blood flow (CBF) and cerebral blood volume (CBV) is vital for furthering our understanding of the BOLD response. The aim of this study was to measure CBF‐CBV coupling in different vascular compartments during neural activation. Three haemodynamic parameters were measured during a visual stimulus. Look‐Locker flow‐sensitive alternating inversion recovery was used to measure changes in CBF and arterial CBV (CBV_a_) using sequence parameters optimized for each contrast. Changes in total CBV (CBV_tot_) were measured using a gadolinium‐based contrast agent technique. Haemodynamic changes were extracted from a region of interest based on voxels that were activated in the CBF experiments. The CBF‐CBV_tot_ coupling constant *α*_tot_ was measured as 0.16 ± 0.14 and the CBF‐CBV_a_ coupling constant *α*_a_ was measured as 0.65 ± 0.24. Using a two‐compartment model of the vasculature (arterial and venous), the change in venous CBV (CBV_v_) was predicted for an assumed value of baseline arterial and venous blood volume. These results will enhance the accuracy and reliability of applications that rely on models of the BOLD response, such as calibrated BOLD.

**Abbreviations used**
ASL: arterial spin labelling
BOLD: blood oxygenation level dependent
CASL: continuous arterial spin labelling
CBF: cerebral blood flow
CBV: cerebral blood volume
EPI: echo planar imaging
GBCA: gadolinium‐based contrast agent
ITS‐FAIR: inflow turbo‐sampling echo planar imaging flow‐sensitive alternating inversion recovery
iVASO: inflow‐based vascular space occupancy
LL‐EPI: Look‐Locker echo planar imaging
LL‐FAIR: Look‐Locker flow‐sensitive alternating inversion recovery
PASL: pulsed arterial spin labelling
PCASL: pseudocontinuous arterial spin labelling
SENSE: sensitivity encoding
SNR: signal to noise ratio
VASO: vascular space occupancy
VENC: velocity encoding
VERVE: venous refocusing for volume estimation

## 1 INTRODUCTION

The relationship between changes in cerebral blood flow (CBF) and cerebral blood volume (CBV) is critical for an accurate understanding of the haemodynamics that underlie blood oxygenation level dependent (BOLD) fMRI, and a greater understanding of cerebral haemodynamics will lead to more precise quantification of the BOLD response.^1^, ^2^ Early studies suggested that the majority of CBV change in response to neuronal activation occurs in venous vessels.^3^, ^4^ This led to the adoption of a CBF‐CBV coupling model based on a power law relationship that was characterized using PET measurements of CBF and total CBV (CBV_tot_).^5^ However, it is now well known that arterial CBV (CBV_a_) also increases on activation, and that this occurs to a much greater degree than total CBV, despite the lower baseline CBV of the arterial compartment.^6^, ^7^, ^8^ Therefore, the use of a CBF‐CBV_tot_ coupling constant will overestimate changes in venous CBV (CBV_v_). This led to measurements of a CBF‐CBV_v_ coupling constant using the CBV_v_ sensitive venous refocusing for volume estimation (VERVE) technique.^9^, ^10^ Despite this, the coupling between CBF and CBV_a_ is still poorly understood in humans, with the majority of research having been performed in rats.^6^, ^7^ Whilst changes in CBV_a_ are typically invisible in the context of standard BOLD fMRI, they become significant in studies utilizing intravascular contrast agents^11^ or hypoxic hypoxia,^12^ where an arterial signal change occurs due to the presence of paramagnetic contrast agent or deoxyhaemoglobin, respectively, in the arterial blood volume, the latter having implications for the application of these methods in cerebrovascular disease, where patients may have a reduced arterial oxygen saturation.

One reason why there is only a small number of published studies examining the relationship between these parameters is the limited number of techniques for measuring CBV_tot_, CBV_a_ and CBV_v_ in humans. Fractional changes in CBV_tot_ (ΔCBV_tot_) during a stimulus have been measured via an infusion of a gadolinium‐based contrast agent (GBCA).^13^, ^14^, ^15^ Such experiments rely on measuring changes in the stimulus evoked BOLD response as a function of intravascular contrast agent concentration, which can either be increased using an extended infusion, or decreased by clearance of a bolus of contrast agent via the kidneys.^16^ This is in contrast to dynamic susceptibility contrast based techniques that are not generally temporally resolved and are more commonly used to measure absolute resting CBV_tot_. Vascular space occupancy (VASO) has also been shown to provide a method for the assessment of total CBV.^17^ However, it is not possible to measure ΔCBV_tot_ using VASO without prior knowledge of the baseline CBV_tot_, thus making this technique unsuitable for the study of CBF‐CBV_tot_ coupling. Arterial spin labelling (ASL)‐based approaches have emerged for the measurement of CBV_a_; these include inflow‐based VASO (iVASO)^18^ and Look‐Locker flow‐sensitive alternating inversion recovery (LL‐FAIR).^8^ LL‐FAIR combines the FAIR ASL technique with Look‐Locker echo planar imaging (LL‐EPI) sampling^19^ to sensitize the signal to either CBF using vascular crushing^20^ or CBV_a_,^8^ depending on the sequence parameters. This technique is also presented in the literature as ITS‐FAIR (inflow turbo‐sampling EPI flow‐sensitive alternating inversion recovery)^21^ and QUASAR (quantitative signal targeting with alternating radio frequency labelling of arterial regions),^22^ the latter requiring the subtraction of scans with and without vascular crushing to estimate CBV_a_. Since the LL‐FAIR technique is also capable of measuring transit time of labelled blood, it is also able to account for changes in transit time that may occur during neuronal activation. LL‐FAIR also has a higher signal to noise ratio (SNR) per unit time for measurement of perfusion than conventional FAIR.^21^ CBV_v_ measurements have been made using the VERVE technique^23^ or hyperoxia BOLD contrast^24^ methods. However, VERVE is hampered by assumptions regarding oxygenation changes during activation and hyperoxia BOLD contrast by relatively low SNR.

In this study, LL‐FAIR‐based measurements of CBF and CBV_a_ are acquired alongside estimates of ΔCBV_tot_ using a GBCA technique. The relationship between the resultant haemodynamic parameters is assessed using a power law relationship, building upon early studies of CBF‐CBV coupling,^5^ the calibrated BOLD method^1^, ^2^ and BOLD modelling studies.^3^, ^4^ This analysis yields CBF‐CBV_a_ and CBF‐CBV_tot_ coupling constants. These measurements are also used to predict the change in CBV_v_ for an assumed arterial and venous volume fraction.

## 2 THEORY

### 2.1 LL‐FAIR for measurement of CBF

**Figure 1:**
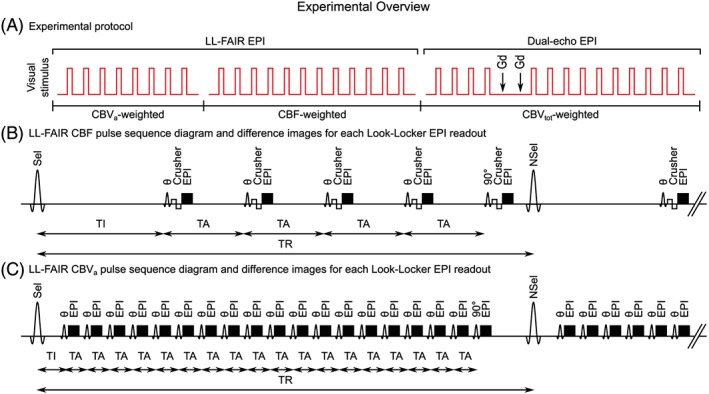
A, overview of the experimental protocol including visual stimulus blocks (red lines), contrast agent injections (arrow Gd) and experimental details. B, example pulse sequence diagram of LL‐FAIR scheme used for CBF quantification with FAIR labelling followed by five EPI readouts. The initial four flip angles have *θ* = 35°, with a final readout pulse of flip angle of 90°. The first readout pulse occurs following an initial inversion delay (*T*_I_ = 600 ms) with subsequent readouts separated by a time interval (*T*_A_ = 350 ms). Data are collected with vascular crushing (VENC = 7.8 mm/s). the sequence is then repeated with repetition time *T*_R_. C, CBV_a_ quantification with FAIR labelling followed by 19 EPI readouts. The first 18 flip angles are *θ* = 50°, with a final readout pulse of flip angle of 90°. The first readout pulse occurs following an initial inversion delay (*T*_I_ = 150 ms), with subsequent readouts separated by a time interval (*T*_A_ = 100 ms). The sequence is then repeated with repetition time *T*_R_

LL‐FAIR combines a FAIR ASL labelling scheme with LL‐EPI sampling. The sequence can be sensitized to CBF by using an initial inversion delay (*T*_I_) to allow inflowing blood to arrive at the imaging plane (*T*_I_ = 600 ms) followed by low flip angle pulses (*θ* = 35°) for the EPI readouts with a time interval (*T*_A_ = 350 ms) such that the perfusion signal is not fully suppressed and can be sampled multiple times Figure 1B). Vascular crushing is added to minimize any CBV_a_ signal contribution from the macrovasculature.

CBF is then quantified by iteratively modelling the signal due to multiple readout pulses using a kinetic model that takes into account the duration of the labelled blood arriving in the tissue, the effect of the readout pulse on both arterial blood magnetization and tissue magnetization, and incomplete blood magnetization recovery at short repetition times. This is performed by solving a three‐compartment model, where Compartment 1 contains arterial blood located outside of the imaging volume, Compartment 2 contains arterial blood inside the imaging volume and Compartment 3 contains blood in the capillary bed, which is in exchange with the tissue. For a complete description see Reference 20.

### 2.2 LL‐FAIR for measurement of CBV_a_

Alternatively, the LL‐FAIR scheme can be sensitized to CBV_a_. This is performed by using an LL‐FAIR scheme with a short initial delay (*T*_I_ = 150 ms) and closely spaced (*T*_A_ = 100 ms) high flip angle pulses (*θ* = 50°) for the EPI readout, which are acquired at the shortest achievable echo time (Figure 1C). This has the advantage of providing a large number of EPI readout time points of high SNR to well sample the arterial blood volume inflow curve for quantification of CBV_a_,^8^ whilst suppressing any CBF contribution.^20^ CBV_a_ is quantified by iteratively modelling the signal across the readout pulses in the arterial blood compartment (Compartment 2 described above) using a kinetic model.^8^

### 2.3 Gadolinium infusion for measurement of CBV_tot_

By infusing a contrast agent during an fMRI paradigm whilst acquiring dual‐echo BOLD weighted images for estimation of the transverse relaxation rate (*R*_2_*), the signal can be sensitized to changes in CBV_tot_. Quantification of this signal is achieved by comparing measurements of *R*_2_* at rest and during activation.^13^, ^14^, ^15^ Under the assumption that stimulus evoked changes in *R*_2_* are constant across multiple trials, additional contributions to *R*_2_* across trials are expected to be solely due to the contrast agent in the blood. In addition, it is assumed that the effect of the contrast agent on the measured signal is predominantly extravascular in origin. Therefore, during rest the measured change in *R*_2_* due to contrast agent can be modelled as

(1) $$\Delta R_{2}^{*\text{rest}}=\kappa \;V_{\mathrm{tot}}\;\left[\mathrm{CA}\right]\;\chi_{\mathrm{CA}},$$

where *κ* is a constant representing physical properties of the experiment, *V*_tot_ is the resting CBV_tot_, [CA] is the contrast agent concentration in the blood and *χ*_CA_ is the molar magnetic susceptibility of the contrast agent.^11^ During the active condition, the measured change in *R*_2_* due to contrast agent is

(2) $$\Delta R_{2}^{*\mathrm{act}}=\kappa \;\left(V_{\mathrm{tot}}+\Delta V_{\mathrm{tot}}\right)\left[\mathrm{CA}\right]\;\chi_{\mathrm{CA}},$$

where Δ*V*_tot_ is the resultant change in CBV_tot_. Therefore, the fractional change in CBV_tot_ (δCBV_tot_) can be estimated by taking the ratio of Equation 1 and Equation 2.

(3) $$\frac{\Delta R_{2}^{*\mathrm{act}}}{\Delta R_{2}^{*\text{rest}}}=\frac{\Delta V_{\mathrm{tot}}}{V_{\mathrm{tot}}}+1=\delta \mathrm{CB}V_{\mathrm{tot}}+1.$$

In an idealized experiment the fMRI stimulus paradigm would be repeated twice, first in the absence of contrast agent and second during a steady state concentration of contrast. However, in practice this is not feasible in human subjects due to dosage limits of GBCAs and their rapid elimination. Therefore, here *R*_2_* measurements are made in the presence of a slowly changing contrast agent concentration provided by an infusion of a GBCA, with $\Delta R_{2}^{*\text{rest}}$ being extrapolated from periods of rest during activated time points.^15^

## 3 METHODS

### 3.1 Imaging

This study was approved by the University of Nottingham Medical School Ethics Committee. Eight healthy volunteers aged 20 to 31 years (24 ± 3 years, mean ± standard deviation) gave written consent and were scanned as part of this study. A schematic diagram of the experimental protocol is given in Figure 1a. Subjects were cannulated prior to entering the scanner to provide access for the injection of contrast agent and ensure that any motion during the Gd injection in the CBV_tot_ experiment was minimal.

Data were acquired on a Philips Achieva 3 T system (Philips Healthcare, Best, The Netherlands), using a body transmit coil and eight‐channel SENSE (sensitivity encoding) head receive coil. All three haemodynamic parameter measurements were acquired with a common spatial resolution of 3 × 3 × 5 mm^3^, matrix size of 64 × 64 and SENSE factor 2 and matched bandwidth. Measurements of CBF and CBV_a_ were limited to a single slice acquisition at the time these experiments were performed. Therefore, an initial functional localizer scan was performed in order to select a single axial slice through the visual cortex with the largest region of BOLD activation. This slice prescription was then used throughout the rest of the experiment.

Visual stimulation was provided by red LED goggles flashing at 8 Hz. Lights were on for 19.2 s and off for the remainder of the 60 s cycle. The number of stimulus cycles varied reflecting the differing SNR of each method: eight cycles were collected for CBV_a_ measurements, 12 cycles for CBF (which has a lower contrast to noise ratio) and 14 cycles for CBV_tot_.

An LL‐FAIR acquisition was used to allow the assessment of both CBF and CBV_a_ measurements accounting for transit time effects. The combination of a Look‐Locker acquisition with a FAIR preparation enables the tagged bolus to be tracked through the macrovascular system and into the tissue sensitizing the signal to CBV_a_ or CBF by using the appropriate combination of sequence parameters. In both cases the thickness of the inversion slab was alternated between 30 mm and 200 mm for label and control conditions, respectively. The sequence parameters for the CBF measurement comprised an initial inversion delay *T*_I_ = 600 ms, time interval between EPI readouts *T*_A_ = 350 ms (resulting in an inversion time range of 600 ms to 2000 ms), flip angle *θ* = 35° and five readout pulses with vascular crushing (bipolar lobe of 5 ms duration per lobe and amplitude of 15 mT, velocity encoding (VENC) = 7.8 mm/s). For the CBV_a_ measurement, the sequence comprised *T*_I_ = 150 ms, *T*_A_ = 100 ms (resulting in an inversion time range of 150 ms to 1950 ms), *θ* = 50°, with 19 readout pulses. In both CBF and CBV_a_ measurements the shortest achievable echo time of 16 ms was used and the final LL‐FAIR pulse had a flip angle of 90° to maximize SNR. The LL‐FAIR scheme was performed with in‐plane pre‐ and post‐saturation pulses to provide signal suppression of the imaging slice, thus reducing any offset signals due to imperfections between the selective and non‐selective RF inversion pulses. The application of a 90° pulse at the end of each *T*_R_ simplified the modelling, as it ensured that each tag/control acquisition is independent, removing the need for an iterative fit of the data to be performed. The *T*_R_ between inversion pulses was 2.4 s, resulting in a label/control pair being collected every 4.8 s.

For the measurement of CBV_tot_, dual‐echo GE‐EPI images were acquired with *T*_E_ = 13/35 ms, *T*_R_ = 1.2 s and three slices. Two single doses (0.2 mL kg^−1^) of Gadoteridol (ProHance, Bracco Imaging, Milan, Italy) were injected, the first bolus at the beginning of the fifth stimulus cycle and the second at the beginning of the sixth cycle. For Cycles 5 and 6, the visual stimulus was not presented and data from these cycles were not used in the estimation of ΔCBV_tot_. The final 10 cycles of the visual stimulus, after contrast agent injection, were acquired at different contrast agent concentration levels during clearance of the contrast by the kidneys.

### 3.2 Analysis

For each subject, CBF, CBV_a_ and CBV_tot_ data sets were first realigned within each data set and then across all data sets using SPM5 (5 mm FWHM Gaussian smoothing kernel and second degree *B*‐spline interpolation).^25^ Since the CBF and CBV_a_ data were acquired for a single slice, motion correction was restricted to in‐plane motion and rotation. In the case of the CBV_tot_ measurement, the acquisition of three slices provided for the correction of small amounts of through‐slice motion. For the CBF and CBV_a_ data, the images from the final LL‐FAIR readout pulse (90° flip angle and thus highest SNR) of each volume acquisition (*T*_R_ period) were realigned, and this transformation matrix then applied to the images acquired from the other LL readout pulses within the corresponding *T*_R_ period. The CBV_tot_ datasets were realigned using the images acquired at the first echo, and this transformation matrix was then applied to the second echo data. Data for each echo time was then down‐sampled to produce a complete time series with a 2.4 s temporal resolution (matching the CBF and CBV_a_ datasets), and only a single slice co‐registered to the CBF and CBV_a_ datasets was retained.

For the CBF and CBV_a_ data, difference images were first computed from the subtraction of consecutive label and control pairs to provide a time series of CBF‐ and CBV_a_‐weighted images for each LL‐readout pulse. Average CBF‐ and CBV_a_‐weighted time series during the visual stimulus cycle were then formed by averaging across cycles, accounting for jittering in the data relative to the stimulus paradigm (thus generating a CBF‐weighted and CBV_a_‐weighted time series of LL readouts for each time point per stimulus cycle). CBV_a_‐weighted difference images were quantified to estimate arterial transit time and CBV_a_ voxelwise using a two‐parameter fit, as described in Reference 8, over an averaged stimulus cycle. CBF‐weighted difference images were initially analysed using a two‐parameter fit for capillary transit time and CBF.^20^ However, since the data had lower SNR than the CBV_a_ data, a two‐parameter fit for each time point within the stimulus cycle was found to increase the noise in the measurement of CBF. Therefore, a mean estimate of the transit time at baseline and on activation was computed and used in a one‐parameter model fit to produce a voxelwise estimates of CBF at each time point over an averaged stimulus cycle.

For the CBV_tot_ data, time series of *R*_2_* values were calculated for each voxel using the realigned, down‐sampled, dual echo data. The fractional change in CBV_tot_ (δCBV_tot_) was calculated by considering the effect of the contrast agent on the *R*_2_* (transverse relaxation rate) changes that occur during the BOLD response, as shown previously by using an infusion to gradually increase the contrast agent concentration.^15^ However, in this study two bolus injections of contrast agent were used to raise the initial blood contrast agent concentration, which gradually decreased due to washout through the kidneys. In the analysis, four stimulus cycles prior to the contrast agent injections provided a baseline. Stimulus cycles following immediately after the injections were discarded to allow for recirculation of the contrast agent, leaving the final nine cycles for analysis. In all other aspects, this method is the same as in previous reports, and resulted in an estimate of ΔCBV_tot_ over an averaged stimulus cycle.^11^, ^15^

Activated regions were generated for each subject using a correlation analysis of the stimulus delivery applied to the quantified CBF maps in order to be maximally sensitive to the site of activation. Each CBF region of interest (ROI_CBF_) was defined based on the CBF statistical map threshold at *p* < 0.01 (uncorrected). In addition, a supplementary analysis was performed to examine the impact of a different ROI definition based on common activated voxels across the three measures of CBV_a_, CBF and δCBV_tot_. Statistical maps from each measure were thresholded at *p* < 0.01 (uncorrected) and voxels at the intersection retained to form a common ROI (ROI_COMMON_).

### 3.3 Estimation of CBF‐CBV coupling

The coupling between CBF and CBV during neuronal activation was calculated assuming a power law relationship between CBF and CBV_tot_,

(4) $$\mathrm{CB}V_{\mathrm{tot}}=\zeta \;\mathrm{CB}F^{\alpha_{\mathrm{tot}}},$$

where values of *ζ* and *α*_tot_ have previously been measured in rhesus monkeys using a hyper/hypocapnia challenge as 0.80 and 0.38, respectively.^5^ Given measurements of the fractional change (i.e. the absolute change in CBV divided by absolute baseline CBV) in CBV_tot_ (δCBV_tot_) and CBF (δCBF) in response to the visual stimulus, which is dimensionless, the CBF‐CBV_tot_ coupling constant *α*_tot_ (Grubb's constant) can be estimated by rearranging Equation 4:

(5) $$\alpha_{\mathrm{tot}}=\frac{\ln\left(\mathrm{\delta CB}V_{\mathrm{tot}}+1\right)}{\ln\left(\text{δCBF}+1\right)}.$$

Under the assumption that the relationship between CBF and CBV_a_ can be described by the power law relationship given in Equation 4, a CBF‐CBV_a_ coupling constant *α*_a_ can be defined. In this case δCBV_a_ is the fractional change in CBV_a_, which is also dimensionless.

(6) $$\alpha_{a}=\frac{\ln\left(\mathrm{\delta CB}V_{a}+1\right)}{\ln\left(\text{δCBF}+1\right)}.$$

Using these equations, Grubb's constant, *α*_tot_, and the CBF‐CBV_a_ coupling constant, *α*_a_, were estimated on a per subject basis. Values for δCBF, δCBV_tot_ and δCBV_a_ were extracted from the quantified maps of each parameter and averaged over each of the ROIs. The time window for the active on condition was defined as between 9.6 s and 19.2 s from the start of the stimulus cycle, whilst the baseline off condition was defined to be between 40.8 s and 60 s. Estimates of *α*_tot_ and *α*_a_ were then computed using Equations 5 and 6, respectively.

In the absence of direct measurements of CBV_v_, we estimated the fractional change in venous CBV (δCBV_v_) using a simple model of the vascular compartments. Changes in total blood volume were approximated as a volume‐weighted sum of two compartments: arterial and venous,

(7) $$\mathrm{\delta CB}V_{\mathrm{tot}}=f_{a}\;\mathrm{\delta CB}V_{a}+f_{v}\mathrm{\delta CB}V_{v},$$

where *f*_a_ and *f*_v_ are the volume fractions assigned to arterial and venous blood volume compartments (*f*_a_ = CBV_a_/CBV_tot_, *f*_v_ = CBV_v_/CBV_tot_), respectively, and here it is assumed that *f*_a_ + *f*_v_ = 1. The capillary volume is assumed to be distributed between the arterial and venous compartments. By rearranging Equation 7, δCBV_v_ can be predicted as a function of arterial and venous volume fractions *f*_a_ and *f*_v_:

(8) $$\mathrm{\delta CB}V_{v}=\frac{\mathrm{\delta CB}V_{\mathrm{tot}}-f_{a}\mathrm{\delta CB}V_{a}}{f_{v}}.$$

## 4 RESULTS

**Figure 2:**
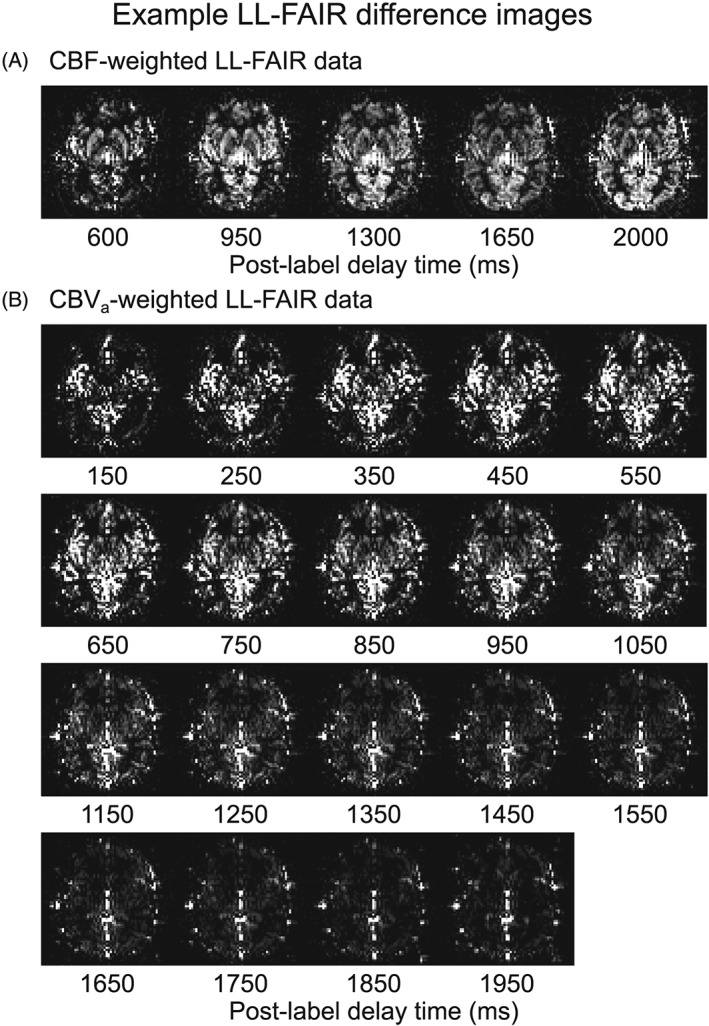
Example LL‐FAIR difference images from a single subject as a function of post‐label delay time averaged over all experimental time points. A, CBF‐weighted images were acquired using five EPI readouts with the first four flip angles of *θ* = 35° and a final excitation pulse of *θ* = 90°. B, CBV_a_‐weighted images were acquired using 19 EPI readouts with flip angles of *θ* = 50° and a final excitation pulse of *θ* = 90°

**Figure 3:**
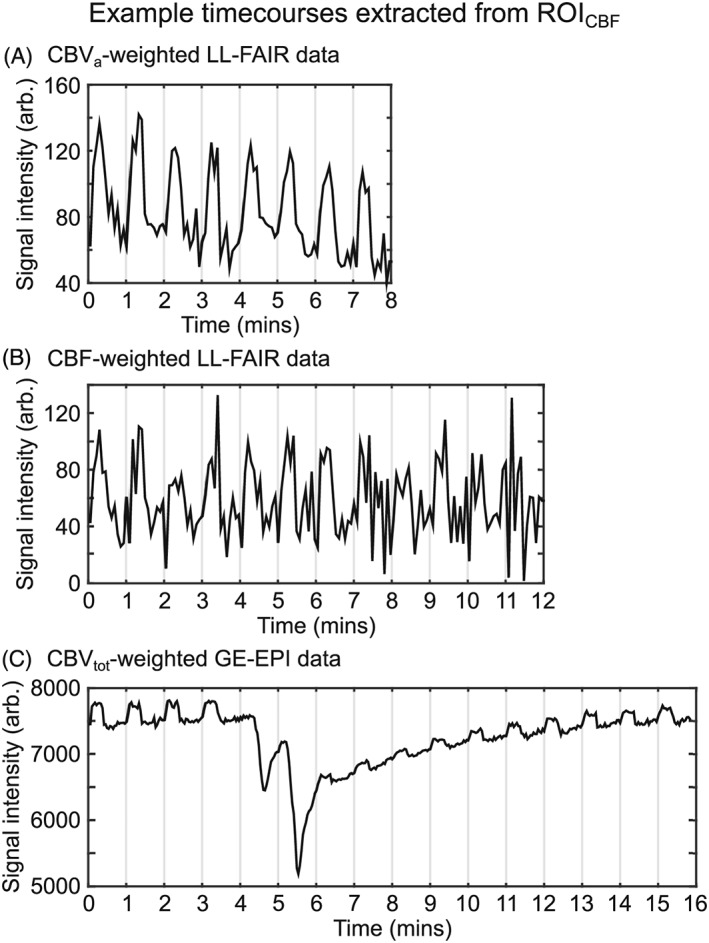
Example time courses of the raw signal for each modality from a single subject averaged over the CBF‐based region of interest. A, CBV_a_‐weighted LL‐FAIR difference data were acquired over eight stimulus cycles. B, CBF‐weighted LL‐FAIR difference data were acquired over 12 stimulus cycles. C, CBV_tot_‐weighted gradient echo (GE) EPI data were acquired over a period of 16 min. Two single‐dose boluses of a GBCA were injected at the beginning of minutes 4 and 5. The visual stimulus was presented for the remaining cycles

Figures 2 and 3 show examples of the raw data acquired in this study from a single subject. The former shows CBF‐weighted (Figure 2A) and CBV_a_‐weighted (Figure 2B) LL‐FAIR difference images as a function of post‐label delay time averaged over all experimental timepoints; note the higher CNR of the CBV_a_‐weighted data. The latter presents time courses of CBV_a_‐, CBF‐ and CBV_tot_‐weighted data averaged over the voxels in the ROI_CBF_ (Figure 3). For this, CBF weighting was achieved by averaging across three LL readouts with post‐label delay times of 950, 1300 and 1650 ms. Similarly, CBV_a_ weighting was obtained by averaging across eight LL readouts with post‐label delay times of 350, 450, 550, 650, 750, 850, 950 and 1050 ms. Finally, the CBV_tot_‐weighted time course was generated using the second echo of the GE‐EPI dataset (*T*_E_ = 35 ms).

Estimates of the mean transit time for CBF‐weighted (perfusion transit time) and CBV_a_‐weighted (arterial transit time) data are presented in Table 1. These data were extracted from the CBF‐based ROI of each subject. During activation the perfusion transit time was found to be significantly reduced (*p* < 0.05, paired two‐tailed *t*‐test), but the change in the arterial transit time was not significant (*p* = 0.11).

**Table 1:** Data extracted from ROI_CBF_. Experimental measurements of the mean CBF‐weighted (perfusion) and CBV_a_‐weighted (arterial) transit times. Per subject standard deviations are presented along with the group mean and standard deviation of each parameter weighted by the number of voxels in each subject's ROI

**Subject no**	**No of voxels**	**Perfusion transit time**		**Arterial transit time**	
		**On**	**Off**	**On**	**Off**
1	22	0.73 ± 0.04	0.72 ± 0.10	0.23 ± 0.08	0.24 ± 0.09
2	41	0.56 ± 0.05	0.66 ± 0.08	0.18 ± 0.01	0.21 ± 0.01
3	80	0.44 ± 0.03	0.61 ± 0.05	0.38 ± 0.01	0.54 ± 0.06
4	5	0.55 ± 0.13	0.46 ± 0.13	0.12 ± 0.02	0.16 ± 0.12
5	70	0.58 ± 0.04	0.80 ± 0.17	0.16 ± 0.01	0.13 ± 0.02
6	107	0.64 ± 0.02	0.67 ± 0.04	0.29 ± 0.02	0.24 ± 0.02
7	77	0.46 ± 0.04	0.66 ± 0.04	0.26 ± 0.01	0.38 ± 0.03
8	13	0.78 ± 0.06	0.93 ± 0.08	0.51 ± 0.06	0.63 ± 0.06
Weighted mean & st. dev.		0.56 ± 0.10	0.68 ± 0.08	0.27 ± 0.08	0.31 ± 0.15

**Figure 4:**
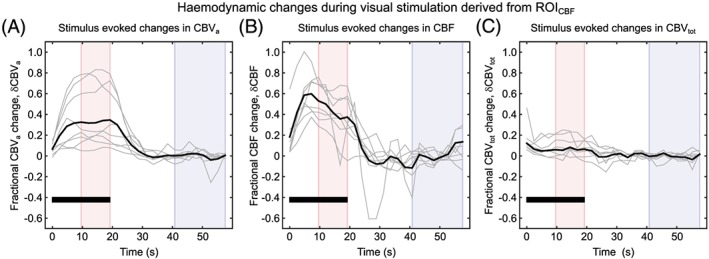
Haemodynamic changes during visual stimulation extracted from an ROI defined based on changes in CBF in response to the stimulus: Fractional change in arterial CBV (δCBV_a_) (a), fractional change in CBF (δCBF) (B) and fractional change in total CBV (δCBV_tot_) (C). time courses are displayed for all subjects (grey lines) and group mean weighted by the number of voxels in each subject's ROI (black solid line). The visual stimulus period is denoted by a solid black bar and averaging windows highlighted for on (pink; 9.6–19.2 s) and off (blue; 40.8–60 s) conditions

Figure 4 displays the time course for the dimensionless fractional change in each haemodynamic measure (δCBV_a_, δCBF and δCBV_tot_) per subject (grey lines), and the group mean weighted by the number of voxels in each subject's CBF derived ROI (solid black line) (see Table 1). Table 2 provides the fractional change measured for each haemodynamic parameter for ROI_CBF_, along with the predicted value of δCBV_v_ estimated assuming a value of arterial volume fraction of *f*_a_ = 0.3 (*f*_v_ = 0.7) (this choice of value is discussed below). The uncertainty in each parameter was estimated as the standard deviation of the voxels in the ROI for the measured parameters (baseline and changes in CBV_a_, CBF and CBV_tot_) and propagated from these values for the calculated parameters (δCBV_v_, *α*_tot_ and *α*_a_). Measurements of δCBV_tot_ and δCBV_a_ were found to be statistically significantly different from zero across the group (one‐sample *t*‐test, *p* < 0.05), with estimates of *α*_tot_ and *α*_a_ provided in Table 2 for each subject and group means of 0.16 ± 0.14 and 0.65 ± 0.24, respectively. In contrast, the estimated value of δCBV_v_ was not statistically significantly different from zero (*p* = 0.33), which would suggest a CBF‐CBV_v_ coupling constant, *α*_v_, of close to zero. Quantified baseline estimates of CBF and CBV_a_ from the baseline time window are also presented in Table 2. Baseline CBV_a_ was found to be inversely correlated with δCBV_a_ (correlation coefficient, *R* = −0.68). Group means and standard deviations in Table 2 are weighted by the number of voxels in each subject's ROI. The number of voxels included in the ROI varied across subjects, probably due to the use of single‐slice techniques, and in some subjects sub‐optimal placement of the slice. It should be noted that, whilst measurements of δCBV_tot_ have the lowest group standard deviation, when coupled with a low mean value of δCBV_tot_ this technique has the highest relative standard deviation. This propagates through to greater uncertainty in the value of *α*_tot_ compared with *α*_a_.

**Table 2:** Data extracted from ROI_CBF_. Experimental measurements of baseline CBF in mL/100 g/min and arterial CBV (CBV_a_) in mL/100 g are presented alongside dimensionless steady state fractional changes in CBF (δCBF), CBV_a_ (δCBV_a_) and CBV_tot_ (δCBV_tot_), as well as values of δCBV_v_ estimated assuming a value of arterial volume fraction *f*_a_ = 0.3 (*f*_v_ = 0.7). Values of the exponents *α*_tot_ and *α*_a_ are calculated on a per subject basis. Per subject standard deviations are presented along with the group mean and standard deviation of each parameter weighted by the number of voxels in each subject's ROI

**Subject**	**No of voxels**	**CBF**	**δCBF**	**δCBV**_**tot**_	**CBV**_**a**_	**δCBV**_**a**_	**δCBV**_**v**_	***α***_**tot**_	***α***_**a**_
1	22	101.7 ± 6.9	0.22 ± 0.16	0 ± 0.03	4.12 ± 0.04	0.06 ± 0.02	−0.03 ± 0.04	−0.02 ± 0.13	0.31 ± 0.20
2	41	66.9 ± 4.9	0.39 ± 0.14	0.06 ± 0.03	4.51 ± 0.03	0.15 ± 0.05	0.02 ± 0.05	0.18 ± 0.10	0.43 ± 0.13
3	80	66.9 ± 2.3	0.37 ± 0.07	0.07 ± 0.02	0.94 ± 0.02	0.26 ± 0.04	−0.02 ± 0.03	0.20 ± 0.06	0.72 ± 0.12
4	5	54.5 ± 4.2	0.63 ± 0.18	0.22 ± 0.04	0.69 ± 0.02	0.77 ± 0.08	−0.02 ± 0.06	0.41 ± 0.11	1.18 ± 0.28
5	70	47.3 ± 11.7	0.49 ± 0.52	−0.01 ± 0.07	1.48 ± 0.19	0.21 ± 0.16	−0.10 ± 0.12	−0.02 ± 0.17	0.47 ± 0.41
6	107	53.3 ± 10.2	0.67 ± 0.28	0.04 ± 0.05	0.79 ± 0.03	0.61 ± 0.07	−0.20 ± 0.08	0.08 ± 0.10	0.94 ± 0.31
7	77	60.1 ± 10.2	0.41 ± 0.09	0.13 ± 0.04	1.76 ± 0.02	0.16 ± 0.03	0.12 ± 0.06	0.37 ± 0.13	0.42 ± 0.07
8	13	73.7 ± 4.4	0.63 ± 0.12	0.19 ± 0.10	0.83 ± 0.04	0.75 ± 0.11	−0.05 ± 0.15	0.36 ± 0.18	1.15 ± 0.17
Weighted mean & st. dev.		60.7 ± 12.3	0.48 ± 0.13	0.06 ± 0.05	1.37 ± 0.75	0.32 ± 0.21	−0.05 ± 0.11	0.16 ± 0.14	0.65 ± 0.24

The results of the supplementary analysis using a common ROI are presented in the Supporting Information. Figure S1 parallels Figure 4, displaying the fractional changes in each of the haemodynamic measures for the common ROI. However, it should be noted that by producing the ROI_COMMON_ as the intersection of changes in CBV_a_, CBF and CBV_tot_ the number of included voxels is low across all subjects and zero for one subject (Table S1). Despite this, larger changes in all haemodynamic parameters were observed compared with the results from ROI_CBF_, and a significant increase in CBV_v_ was detected (one‐sample *t*‐test, *p* < 0.05). This enabled *α*_v_ to be estimated as 0.29 ± 0.15 across the group, alongside values of 0.45 ± 0.14 for *α*_tot_ and 0.70 ± 0.29 for *α*_a_. These results can be found in Table S2.

Details of how to access the data underpinning the results presented in this study can be found in the appendix.

## 5 DISCUSSION

A good understanding of the relationship between changes in CBF and CBV is important for interpreting the physiological changes that underlie functional hyperaemia. However, how these changes translate to a measured BOLD response depends on how such changes are distributed across the different vascular compartments. The results of this study will help to improve models of the BOLD response.^11^, ^26^ In turn this will enhance the accuracy and reliability of applications that rely on a correct understanding of the BOLD response. For example, it has been shown that the accuracy of the calibrated BOLD method for quantifying stimulus evoked oxygen metabolism changes is critically dependent on accurate knowledge of CBF‐CBV_v_ coupling.^26^, ^27^

In this study, measurements of CBF, CBV_a_ and CBV_tot_ were combined to assess the coupling of CBF changes with changes in arterial, venous and total CBV, within two functionally defined ROIs. For the CBF derived ROI, the CBF‐CBV_tot_ coupling constant *α*_tot_ was estimated to be 0.16 ± 0.14 and the CBF‐CBV_a_ coupling constant *α*_a_ was estimated to be 0.65 ± 0.24 (mean ± standard deviation). The estimated change in CBV_v_ within this ROI was not statistically significant. Supplementary analysis using a common ROI (ROI_COMMON_) provided contrasting results, which are discussed later in this section.

### 5.1 Comparison with the literature

The coupling between CBF and CBV_tot_ has been the target of numerous studies. The power law relationship (Equation 1) was first introduced by Grubb et al, who measured *α*_tot_ to be 0.38 in anaesthetized rhesus monkeys during steady state hyper/hypocapnic challenges.^5^ Further PET measurements in humans, using a combination of radiolabelled water (H_2_^15^O) and carbon monoxide (C^15^O or ^11^CO), measured *α*_tot_ to be 0.3 in response to a visual stimulus^28^ and 0.29 and 0.64 ± 0.26 during a hyper/hypocapnic challenge,^29^, ^30^ respectively (errors reported where available). MRI‐based experiments using a perfluorocarbon contrast agent and continuous ASL (CASL) measured a value of *α*_tot_ of 0.4 in rats.^6^ However, in all of these cases measurements were made in the steady state. In this study, a relatively short stimulus duration of 19.2 s was used, which is more relevant for human imaging studies but may not be long enough to allow a steady state to be reached, and hence may explain the smaller value of *α*_tot_ measured (0.16 ± 0.14) for ROI_CBF_. A larger value of *α*_tot_ was measured (0.45 ± 0.14) for ROI_COMMON_, albeit for a much smaller active region of tissue.

The literature regarding the coupling of CBF with CBV_a_ is more limited. Perfluorocarbon studies on rats measured the CBV_a_ change due to steady state hypercapnia and the change in CBF using CASL.^6^ Reviewing their data, we estimate *α*_a_ = 0.84. A similar rat study performed by Kim et al^7^ measured changes in CBF and CBV_a_ in response to a 15 s somatosensory stimulus using the MOTIVE (MRI with modulation of tissue and vessel) technique,^31^ and from these datasets we estimate *α*_a_ = 1.73. The values for *α*_a_ obtained in our study (0.65 ± 0.24 and 0.70 ± 0.29) are at the low end of this range, again perhaps suggesting that a steady state was not achieved using a short stimulus. However, further data are required to investigate the effects of stimulus, species and anaesthesia differences.

This experiment did not allow a direct measurement of CBV_v_ changes. In addition, it was not possible to acquire absolute estimates of CBV_tot_. Therefore, the changes in CBV_v_ could only be investigated based on assumptions regarding the partitioning of blood volume by a two‐compartment model consisting of arterial and venous volume fractions. The arterial volume fraction *f*_a_ has previously been measured as 0.27,^7^ 0.29,^32^ and 0.3–0.37,^33^ using a range of techniques, each of which is expected to include a small fraction of the post‐arterial capillary blood volume, whilst the venous volume fraction *f*_v_ has been measured as 0.77 using the quantitative BOLD technique.^34^ This technique is specifically sensitive to blood vessels containing deoxygenated blood. Whilst this largely consists of blood within venous vessels, it is also expected to include deoxygenated blood within the capillaries. Therefore, the arterial and venous compartments defined in this study might more accurately be described as the oxygenated and deoxygenated compartments. Importantly, it is this deoxygenated blood volume that underlies the BOLD response and best reflects the BOLD specific CBF‐CBV coupling. Therefore, based on the literature values above, *f*_a_ was assumed to take a value of 0.3. Under this assumption, a non‐significant change in CBV_v_ was estimated for ROI_CBF_, whilst a significant change was recorded for ROI_COMMON_. This would suggest a CBF‐CBV_v_ coupling constant, *α*_v_, of around zero for the former and *α*_v_ = 0.32 ± 0.14 for the latter. These values bracket the limited range of values of CBF‐CBV_v_ coupling constant given in the literature. In rats a value of *α*_v_ of 0.20 was measured using a steady state hypercapnia stimulus,^6^ whilst in humans *α*_v_ was measured as 0.18 for a steady state hyper/hypocapnic stimulus,^10^ and 0.18/0.31 for low/high intensity visual stimulation.^9^ However, even in the latter visual stimulus experiments, the stimulus duration was 96 s and therefore significantly longer than that used in our study. In order to examine the effect of short stimuli we can consider recent work investigating the temporal dynamics of CBV_v_.^35^ In this work CBV_v_ was shown to be delayed with respect to changes in CBF and CBV_a_. Given the short duration of our stimulus, it is likely that this would not produce an appreciable increase in CBV_v_, consistent with the results from ROI_CBF_. Furthermore this is consistent with two photon microscopy measurements that demonstrated a minimal increase in CBV_v_ for a 10 s stimulus, but a larger and delayed response for a 30 s stimulus.^36^ Given these findings, the results from ROI_COMMON_ appear to overestimate the change in CBV_v_.

The inconsistency between the two ROIs considered in this study results from the different voxel selection methods. In the case of ROI_CBF_, the use of a CBF localizer provides sensitivity to perfusion and hence to exchange at the capillary bed. CBF ROIs have previously been shown to be more robust than a BOLD‐based localizer.^37^ In contrast the CBV localizers used in combination with the CBF localizer to define the ROI_COMMON_ are not specific to any particular vessel scale. Therefore, large vessels in the ROI are likely to be accompanied by small changes in CBV, whilst small vessels might see larger changes. Whilst the correlation between baseline CBV_a_ and δCBV_a_ is not statistically significant, this effect likely explains the intersubject variation in the δCBV_a_ time courses (Figures 4 and S1). In addition, it has been observed that changes in CBV_v_ during activation are spatially heterogenous with both increases and decreases.^24^ Therefore, selecting for only positive changes in CBV has the potential to overestimate the fractional change during activation and may explain why a significant change in CBV_v_ was observed for ROI_COMMON_.

### 5.2 Limitations of the current study

In this work, ROIs were selected using the activated region defined by the CBF data. However, the use of such functionally defined ROIs can lead to statistical bias, resulting in an overestimation of changes in CBF.^38^ Due to the importance of maintaining comfort in human volunteer studies, time within the scanner was limited. Therefore, it was not possible to acquire an additional CBF dataset to provide an independent definition of the ROI.

A pulsed ASL (PASL) preparation was employed in this study so that the same methodologies could be used for the quantification of both CBF and CBV_a_ and the effects of transit time could be measured and accounted for. The use of a pseudocontinuous ASL (PCASL) approach to measure changes in CBF would likely improve the fidelity of such measurements. However, a PCASL preparation cannot be used for CBV_a_ measurements due to SAR limitations. Therefore, in practice using a PASL preparation for both CBF and CBV_a_ measurements is preferable to maintain the same labelling efficiency in both experiments.

Absolute quantification of CBV_tot_ was not possible in this study, so we could not measure ΔCBV_v_. This might be measured by tracking the first bolus of contrast agent.^39^ However, the resolution of the data acquired in this study was too coarse to yield an adequate arterial input function. Absolute CBV_tot_ is reported to be in the range of 3–5 mL/100 g, therefore the results of this study would predict an absolute change in CBV_tot_ (ΔCBV_tot_) between 0.18 and 0.3 mL/100 g.

Predictions of *α*_v_ were based on a two‐compartment model of the vasculature: arterial and venous. It was therefore assumed that the capillary compartment was distributed between these two compartments. However, it is likely that changes in capillary CBV will have their own characteristic coupling to CBF and may therefore contribute to an under/overestimation of CBV change in the arterial and venous compartments.

Finally, only steady state changes in haemodynamics were studied since the SNR was not sufficient to study dynamic changes. However, it has been shown that a post‐stimulus undershoot in CBF may contribute to the BOLD post‐stimulus undershoot^40^ and that changes in CBV_v_ are delayed with respect to changes in CBV_a_.^35^ These dynamic variations are expected to add to the complexity of the temporal characteristics of the BOLD response. Repeating this work at higher field might allow the study of these dynamic characteristics, greater knowledge of which may enable the dynamics of changes in oxygen metabolism to be investigated using extensions to methods such as calibrated BOLD.^1^, ^2^

## 6 CONCLUSION

In this study, measurements of CBF, CBV_a_ and CBV_tot_ were performed in individual subjects in a single experimental session to assess the coupling of haemodynamic responses. This information is valuable for furthering our understanding of the BOLD response and for enhancing the accuracy and reliability of applications that rely on models of the BOLD response, such as calibrated BOLD.

## Supporting information


**TABLE S1** Voxel count for ROI_COMMON_. Number of voxels in activated regions of interest (ROI) for cerebral blood flow (CBF), arterial cerebral blood volume (CBV_a_), total cerebral blood volume (CBV_tot_) and the common ROI for individual subjects obtained for z‐score of 2.31. A common ROI was not found for subject 8.
**TABLE S2** Data extracted from ROI_COMMON_. Experimental measurements of baseline cerebral blood flow (CBF) in ml/min/100 g and arterial cerebral blood volume (CBV_a_) in ml/100 g are presented alongside dimensionless steady state fractional changes in CBF (δCBF), CBV_a_ (δCBV_a_) and CBV_tot_ (δCBV_tot_). Values of the exponents αtot and αa are calculated on a per subject basis. Per subject standard deviations are presented for directly measured parameters along with the group mean and standard deviation of each parameter weighted by the number of voxels in each subject's ROI.
**FIGURE S1** Haemodynamic changes during visual stimulation extracted from an ROI defined based on voxels that show significant changes in CBF, CBV_a_ and CBV_tot_: (a) fractional change in arterial cerebral blood volume (δCBV_a_), (b) fractional change in cerebral blood flow (δCBF) and (c) fractional change in total cerebral blood volume (δCBV_tot_). Timecourses displayed for all subjects (grey lines) and group mean weighted by the number of voxels in each subject's ROI (black solid line). The visual stimulus period is denoted by a solid black bar and averaging windows highlighted for ON (pink) and OFF (blue) conditions.

## APPENDIX 1

The data that underpin the results presented in this work can also be accessed via the Zenodo repository (https://doi.org/10.5281/zenodo.1464828).
