# Supplementary material for: Coupling between cerebral blood flow and cerebral blood volume: Contributions of different vascular compartments
Source: NMR Biomed. 2019 Jan 18;32(3):e4061. doi: 10.1002/nbm.4061 (PMC6492110; doi:10.1002/nbm.4061)
Supplement: Supplementary file 1 — TABLE S1 Voxel count for ROICOMMON. Number of voxels in activated regions of interest (ROI) for cerebral blood flow (CBF), arterial cerebral blood volume (CBVa), total cerebral blood volume (CBVtot) and the common ROI for individual subjects obtained for z‐score of 2.31. A common ROI was not found for subject 8. TABLE S2 Data extracted from ROICOMMON. Experimental measurements of baseline cerebral blood flow (CBF) in ml/min/100 g and arterial cerebral blood volume (CBVa) in ml/100 g are presented alongside dimensionless steady state fractional changes in CBF (δCBF), CBVa (δCBVa) and CBVtot (δCBVtot). Values of the exponents αtot and αa are calculated on a per subject basis. Per subject standard deviations are presented for directly measured parameters along with the group mean and standard deviation of each parameter weighted by the number of voxels in each subject's ROI. FIGURE S1 Haemodynamic changes during visual stimulation extracted from an ROI defined based on voxels that show significant changes in CBF, CBVa and CBVtot: (a) fractional change in arterial cerebral blood volume (δCBVa), (b) fractional change in cerebral blood flow (δCBF) and (c) fractional change in total cerebral blood volume (δCBVtot). Timecourses displayed for all subjects (grey lines) and group mean weighted by the number of voxels in each subject's ROI (black solid line). The visual stimulus period is denoted by a solid black bar and averaging windows highlighted for ON (pink) and OFF (blue) conditions. [file NBM-32-na-s001.docx]

**SUPPLEMENTARY MATERIAL**

A supplementary analysis was performed to examine the impact of a different region of interest (ROI) definition. In contrast to the ROI defined purely based on changes in cerebral blood flow (CBF) named ROI_CBF_, this alternative ROI was based on commonly activated voxels (ROI_COMMON_) based on changes in CBF, arterial cerebral blood volume (CBV_a_) and total cerebral blood volume (CBF_tot_). The numbers of voxels selected for each of these haemodynamic measures are listed in Table S1, as well as how many voxels were common to all three modalities.

The procedures to generate Table 2 and Fig. 4 were repeated for data extracted from ROI_COMMON_. Larger changes in all three haemodynamic measurements were found (Fig. S1). This resulted in a larger estimate of α_tot_ than for ROI_CBF_, whilst the group mean estimate of α_a_ was relatively unchanged (Table S2). Given the unchanged relationship between δCBF and δCBV_a_ and the larger δCBV_tot_ response, a significant change in CBV_v_ was estimated (one-sample t-test, p<0.05). It was therefore possible to estimate α_v_ for this ROI, unlike ROI_CBF_. Quantified baseline estimates of CBF and CBV_a_ from the baseline time window are also presented in Table S2. In common with ROI_CBF_, baseline CBV_a_ was found to be inversely correlated with δCBV_a_ (correlation coefficient, R=‑0.61). Group means and standard deviations in Table S2 are weighted by the number of voxels in each subject’s ROI.

**TABLE S1** Voxel count for ROI_COMMON_. Number of voxels in activated regions of interest (ROI) for cerebral blood flow (CBF), arterial cerebral blood volume (CBV_a_), total cerebral blood volume (CBV_tot_) and the common ROI for individual subjects obtained for z-score of 2.31. A common ROI was not found for subject 8.

| **Subject** | **CBV_a_** | **CBF** | **CBV_tot_** | **Common ROI** |
| --- | --- | --- | --- | --- |
| 1 | 8 | 22 | 42 | 1 |
| 2 | 35 | 41 | 21 | 6 |
| 3 | 19 | 80 | 104 | 6 |
| 4 | 12 | 5 | 11 | 2 |
| 5 | 23 | 70 | 10 | 1 |
| 6 | 33 | 107 | 45 | 9 |
| 7 | 60 | 77 | 22 | 10 |
| 8 | 5 | 13 | 21 | 0 |

**FIGURE S1**  Haemodynamic changes during visual stimulation extracted from an ROI defined based on voxels that show significant changes in CBF, CBV_a_ and CBV_tot_: (a) fractional change in arterial cerebral blood volume (δCBV_a_), (b) fractional change in cerebral blood flow (δCBF) and (c) fractional change in total cerebral blood volume (δCBV_tot_). Timecourses displayed for all subjects (grey lines) and group mean weighted by the number of voxels in each subject’s ROI (black solid line). The visual stimulus period is denoted by a solid black bar and averaging windows highlighted for ON (pink) and OFF (blue) conditions.

**TABLE S2** Data extracted from ROI_COMMON_. Experimental measurements of baseline cerebral blood flow (CBF) in ml/min/100g and arterial cerebral blood volume (CBV_a_) in ml/100g are presented alongside dimensionless steady state fractional changes in CBF (δCBF), CBV_a_ (δCBV_a_) and CBV_tot_ (δCBV_tot_). Values of the exponents αtot and αa are calculated on a per subject basis. Per subject standard deviations are presented for directly measured parameters along with the group mean and standard deviation of each parameter weighted by the number of voxels in each subject’s ROI.

| **Subject** | **CBF** | **δCBF** | **δCBV_tot_** | **CBV_a_** | **δCBV_a_** | **δCBV_v_** | **α_tot_** | **α_a_** | **α_v_** |
| --- | --- | --- | --- | --- | --- | --- | --- | --- | --- |
| 1 | 91.5±14.7 | 0.38±0.31 | 0.33±0.08 | 3.01±0.28 | 0.90±0.25 | 0.08±0.16 | 0.87±0.64 | 1.98±1.38 | 0.25±0.48 |
| 2 | 59.4±6.3 | 0.61±0.19 | 0.11±0.04 | 3.78±0.20 | 0.20±0.08 | 0.08±0.07 | 0.23±0.10 | 0.38±0.10 | 0.16±0.15 |
| 3 | 51.3±10.1 | 0.98±0.50 | 0.36±0.08 | 3.93±0.27 | 0.71±0.14 | 0.21±0.12 | 0.45±0.18 | 0.79±0.29 | 0.28±0.18 |
| 4 | 35.5±7.6 | 0.47±0.35 | 0.22±0.10 | 8.62±0.28 | 0.41±0.06 | 0.14±0.15 | 0.51±0.38 | 0.88±0.54 | 0.34±0.40 |
| 5 | 73.6±17.3 | 0.94±0.48 | 0.24±0.12 | 2.02±0.28 | 0.89±0.29 | -0.03±0.21 | 0.33±0.19 | 0.96±0.36 | -0.05±0.33 |
| 6 | 80.8±7.9 | 0.62±0.16 | 0.22±0.04 | 6.64±0.31 | 0.40±0.07 | 0.15±0.06 | 0.42±0.11 | 0.70±0.14 | 0.29±0.13 |
| 7 | 96.8±7.5 | 0.52±0.14 | 0.26±0.10 | 5.73±0.26 | 0.31±0.07 | 0.24±0.15 | 0.55±0.23 | 0.65±0.14 | 0.51±0.31 |
| 8 | - | - | - | - | - | - | - | - | - |
| Weighted group mean ± S.D. | 74.2±19.5 | 0.65±0.17 | 0.24±0.07 | 5.30±1.52 | 0.42±0.20 | 0.17±0.07 | 0.44±0.13 | 0.70±0.27 | 0.32±0.14 |
